# Knotting of a Urinary Catheter and Ureteric Stent: A Unique Complication and Management Solution

**DOI:** 10.1155/criu/5559138

**Published:** 2025-07-10

**Authors:** H. Logan, K. Lockhart, P. Chong

**Affiliations:** ^1^General Surgical Department John Hunter Hospital, HNE Health, Newcastle, Australia; ^2^Urology Department, Royal North Shore Hospital, North Sydney Local Health District, Sydney, Australia; ^3^Urology Department, John Hunter Hospital, Hunter New England Local Health District, Newcastle, Australia

**Keywords:** catheter complication, indwelling catheter, knotting, ureteric stent complication, urinary catheter

## Abstract

**Objective:** Spontaneous intravesical knotting is a highly infrequent complication of urinary catheters. We present a novel endoscopic treatment approach to managing a spontaneously knotted urinary catheter around a ureteric stent.

**Subject:** A 79-year-old man presented to the Emergency department with confusion and acute renal failure. His background was significant for metastatic castrate-resistant prostate cancer. His associated obstructive uropathy was managed with a long-term right 7-Fr Rüsch ureteric stent, last changed 1 month prior and a long-term 18-Fr indwelling catheter. A CT intravenous pyelogram clearly demonstrated his indwelling catheter knotted around and through the distal intravesical portion of an appropriately positioned right ureteric stent.

**Results:** Following decompression of the left kidney via percutaneous nephrostomy, attempts were made to remove the urinary catheter under fluoroscopy with a variety of wires and introducers. The patient then underwent a general anesthesia, and the knot was successfully removed piecemeal with a Mauermayer stone crusher via 25-Fr access sheath.

**Conclusion:** Endoscopic techniques such as the use of a stone crusher may be beneficial for the removal of difficult and complex catheter knots as demonstrated in this case. Catheter knotting should always be considered if the functioning or attempted removal of the catheter is abnormal and timely referral to a urologist is made.

## 1. Introduction

Long-term indwelling urinary catheters are a vital resource in the management of urinary outflow obstruction. They are associated with a variety of complications that may require urological input. One of the most infrequent complications encountered is spontaneous intravesical knotting of these devices. The incidence of this issue has been previously reported to be around 0.2 in 100,000 [1]. Here, we present an endoscopic treatment approach to managing a spontaneously knotted urinary catheter around a ureteric stent. No other published cases were found to document the use of this technique in a relevant literature review.

## 2. Case Presentation

A 79-year-old man presented to the emergency department with confusion and acute renal failure.

His background was significant for metastatic castrate-resistant prostate cancer treated previously with radical prostatectomy, radiotherapy, and chemotherapy with Docetaxel. His associated obstructive uropathy was managed with a long-term right 7-Fr 24 cm Rüsch ureteric stent, last changed 1 month prior, and a long-term 18-Fr indwelling catheter.

Other significant medical history included rectal cancer, ischaemic heart disease, and previous DVT for which he was anticoagulated on Apixaban. On examination, the patient had a low-grade tachycardia and borderline systolic blood pressure of 100 mmHg. There was mild tenderness in the left upper quadrant and left flank, but his abdomen was soft. His catheter was draining small amounts of concentrated urine freely. His renal function was significantly impaired, with a creatinine of 394 mg/dL and eGFR of 12 mL/min (baseline values were around creatinine100 mg/dL and eGFR 60 mL/min, respectively) and CRP was elevated at 120 mg/L. Urinalysis was positive for leukocytes, nitrites, and blood, and later final urine culture demonstrated mixed growth of organisms with urine white cells > 100 (x 10^6^/L).

A CT intravenous pyelogram demonstrated an appropriately positioned right ureteric stent with contrast draining into the bladder and new left hydroureteronephrosis down to the level of the bladder. It also clearly demonstrated his indwelling catheter knotted around and through the distal intravesical portion of the right ureteric stent.

His urosepsis was managed empirically with ampicillin and gentamicin. Left-sided decompression was achieved in the context of sepsis and difficult retrograde access with a left percutaneous nephrostomy, which was completed that evening without complication.

Two days later, once stabilized, an attempt was made in fluoroscopy by the urologist and interventional radiologist to straighten the catheter knot to facilitate catheter removal through a range of techniques. This included the insertion of a variety of wires and a gentle introducer insertion via the catheter lumen, but these attempts were unsuccessful.

He then proceeded to examination under general anesthesia; the catheter was cut as proximal as possible and the distal portion was removed. A cystoscopy was then completed, demonstrating very small bladder capacity and persistent bleeding. The catheter knot was unable to be detangled endoscopically; attempts to break apart the knot with a Collins knife and 24-Fr sheath resectoscope were made difficult by poor vision due to contact bleeding. The knot was successfully removed piecemeal with a Mauermayer stone crusher via 25-Fr access sheath (26-Fr access sheath insertion was not feasible due to a tight rigid bladder neck). The right-sided ureteric stent was exteriorized and exchanged for a new 7-Fr 24-cm Rüsch stent. A 20-Fr suprapubic catheter was inserted. The position of the stent and catheter was confirmed with an image intensifier and cystogram. A left nephrostogram was performed which demonstrated appropriate positioning of the nephrostomy within the collecting system. Filling defects consistent with clotted blood were visualized within the renal pelvis and proximal ureter. These were cleared with saline flushing and, following this, contrast was seen to be passing freely into the bladder. The left nephrostomy was left in situ following the procedure.

**Figure 1:**
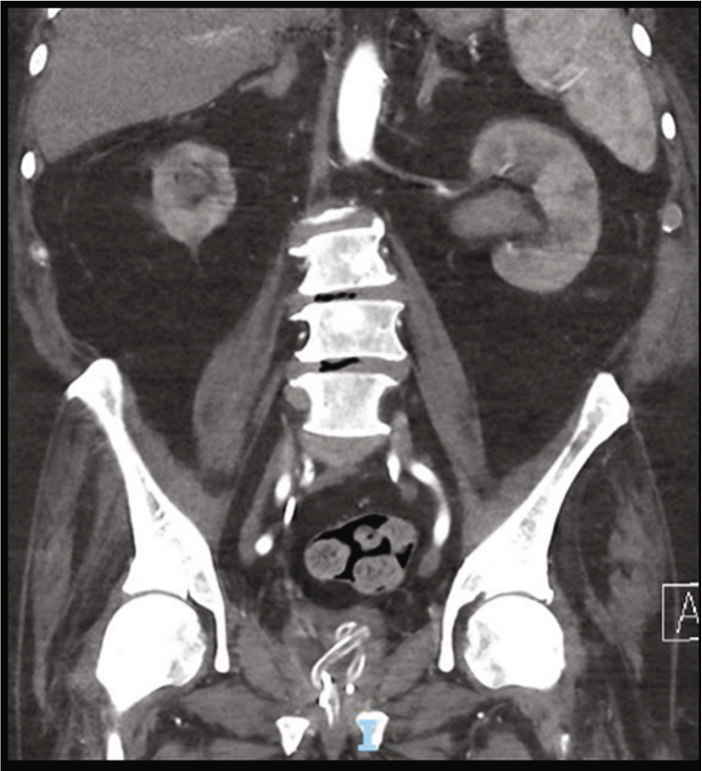
Coronal CT demonstrating knotted catheter and right ureteric stent.

**Figure 2:**
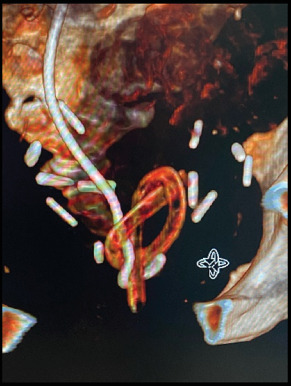
CT 3D reconstructed images of catheter/stent knot.

**Figure 3:**
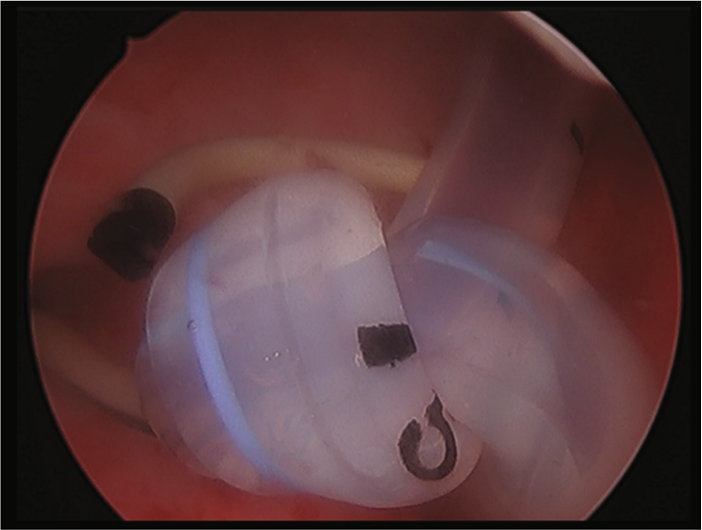
Endoscopic picture of intravesical knot.

**Figure 4:**
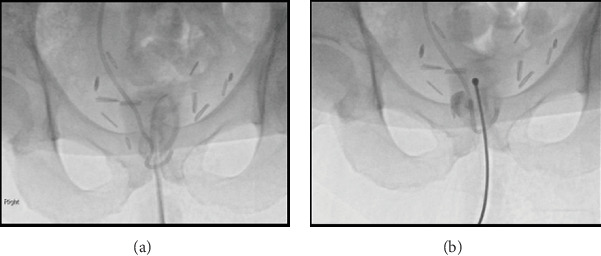
(a, b) Interventional radiology/urology fluoroscopy suite X-ray of the knot and attempt to straighten the knot with introducer following wire attempt.

**Figure 5:**
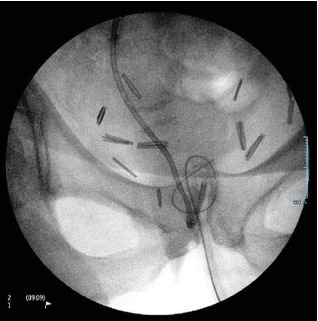
Intraoperative X-ray demonstrating maximal sensor wire insertion.

**Figure 6:**
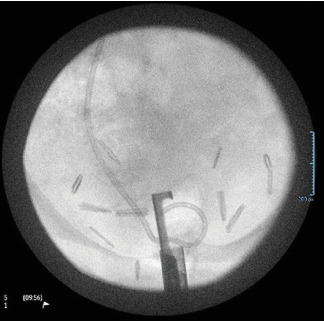
X-ray of Mauermayer stone crusher catheter extraction.

After a thorough discussion with the patient and his family following this procedure, it was determined that his medical comorbidities, significant surgical risk, and overall frailty rendered him unsuitable for further invasive management. Patient quality of life and symptomatic control were prioritized. The main ongoing issues following this procedure included refractory transfusion-dependent anemia and fluctuating levels of consciousness. A referral to the palliative care team was made, and the patient was eventually transferred to a private hospital for ongoing symptomatic management and comfort level of care. Collation of associated radiographic and endoscopic images with descriptions is provided in Figures 1, 2, 3, 4, 5, and 6.

## 3. Discussion

Intravesical catheter knotting is an often technically challenging complication. The mechanism of knotting is likely due to excessive catheter coiling; as the bladder decompresses, the catheter tip can migrate through a coil, forming a true knot [2], often also compounded by a small capacity bladder, forcing the tip to bend or coil even with correct positioning and no undue length within the bladder. A formed knot will then tighten at the internal urethral office as sustained traction is applied during removal.

Previous reports almost exclusively involve a paediatric population, thus smaller calibre catheters and smaller bladder size (proportional to the catheter used) are potentially predisposing factors [3]. Excessive length of catheter insertion, catheter pliability, and bladder spasm may also predispose to intravesical coiling.

The introduction of another catheter to the bladder lumen, in this case a ureteric stent, could increase the chance of these phenomena occurring by providing additional length or as a pivot point for a coil to occur around. Although this scenario is far too rare to draw any definitive conclusions from, a description of urinary catheter knotting around a double-J ureteral stent likely draws the most similarities to this case study in current literature [4].

Due to the rarity of this complication, there remains a reduced awareness within the medical community. Spontaneous knotting should always be considered a possibility if resistance occurs during the standard removal of any urinary catheter.

Avoiding or limiting modifiable risk factors to this problem will further limit this complication which is already rare. Modifiable risk factors include excessive catheter length in the bladder, underinflated or small balloon volumes (<10 mL), high catheter mobility due to inadequate securement or patient agitation, use of softer or more flexible catheter materials, prolonged indwelling time, and improper insertion technique (e.g., forced advancement against resistance). It is also important that specialist advice is sought before any traction is put on a catheter which is not able to be removed normally. Excessive traction is likely to not only tighten the knot but also introduce the risk of damaging internal structures.

Several successful reported solutions for this issue have been discussed in previous literature. In certain populations, successful removal has been achieved simply by gentle continuous traction on the catheter under local or general anesthesia. The only reported sequelae are transient and self-limiting haematuria [5]. Guide-wire–assisted fluoroscopy has been used to untie the knot from within the catheter. This approach aims to straighten the knot from within by increasing the rigidity of the device [6]. Suprapubic open cystostomy catheter removal remains a last resort in scenarios which have failed less invasive management.

## 4. Conclusion

Endoscopic techniques such as the use of a stone crusher may be beneficial for the removal of difficult and complex catheter knots, as demonstrated in this case. Catheter knotting should always be considered if the function or removal of the catheter is abnormal, and a timely referral to a urologist should be made.

## Data Availability

Data sharing is not applicable to this article as no datasets were generated or analyzed during the current study.

## Nomenclature

Fr: French
DVT: deep vein thrombosis
eGFR: estimated glomerular filtration rate
CRP: C-reactive protein
CT: computed tomography

## References

[1] Foster H., Ritchey M., Bloom D. (1992). Adventitious Knots in Urethral Catheters: Report of 5 Cases. *Journal of Urology*.

[2] Mayer E., Ankem M. K., Hartanto V. H., Barone J. G. (2002). Management of Urethral Catheter Knot in a Neonate. *Canadian Journal of Urology*.

[3] Sinha S., Singh V. (2019). Spontaneous Knotting of Urinary Catheters Placed With Nonindwelling Intent: Case Series and Literature Review. *Urology Annals*.

[4] Warmerdam E. G., Toorop R. J., Abrahams A. C., Berger P. (2011). An Indwelling Urethral Catheter Knotted Around A Double-J Ureteral Stent: An Unusual Complication After Kidney Transplantation. *Case Reports in Nephrology*.

[5] Blas L., Roberti J., Lopez F., Ameri C. A. (2021). Water Knot: Case Report of a Rare Complication During a Urodynamic Test. *Journal of Clinical Urology*.

[6] Yiğiter M., Salman A. B. (2016). Intravesical Catheter Knotting: An Unusual Complication of Suprapubic Catheterization. *Turkish Journal of Pediatrics*.

